# Gene model for the ortholog of *eIF4E1* in *Drosophila ananassae*

**DOI:** 10.17912/micropub.biology.001026

**Published:** 2025-01-29

**Authors:** McKenzie Chamberlain, Ali Christie, Jeremy Girard, Hannah M. Shaver, Lindsey J. Long, James J. Youngblom, Chinmay P. Rele, Laura K Reed

**Affiliations:** 1 University of Alabama, Tuscaloosa, Alabama, United States; 2 Oklahoma Christian University, Edmond, Oklahoma, United States; 3 California State University, Stanislaus, Turlock, California, United States

## Abstract

Gene model for the ortholog of eukaryotic translation initiation factor 4E1 ( *eIF4E1* ) in the May 2011 (Agencourt dana_caf1/DanaCAF1) Genome Assembly (GenBank Accession: GCA_000005115.1 ) of *Drosophila ananassae* . This ortholog was characterized as part of a developing dataset to study the evolution of the Insulin/insulin-like growth factor signaling pathway (IIS) across the genus *Drosophila* using the Genomics Education Partnership gene annotation protocol for Course-based Undergraduate Research Experiences.

**Figure 1. Genomic neighborhood and gene model for eIF4E1 in Drosophila ananassae:**
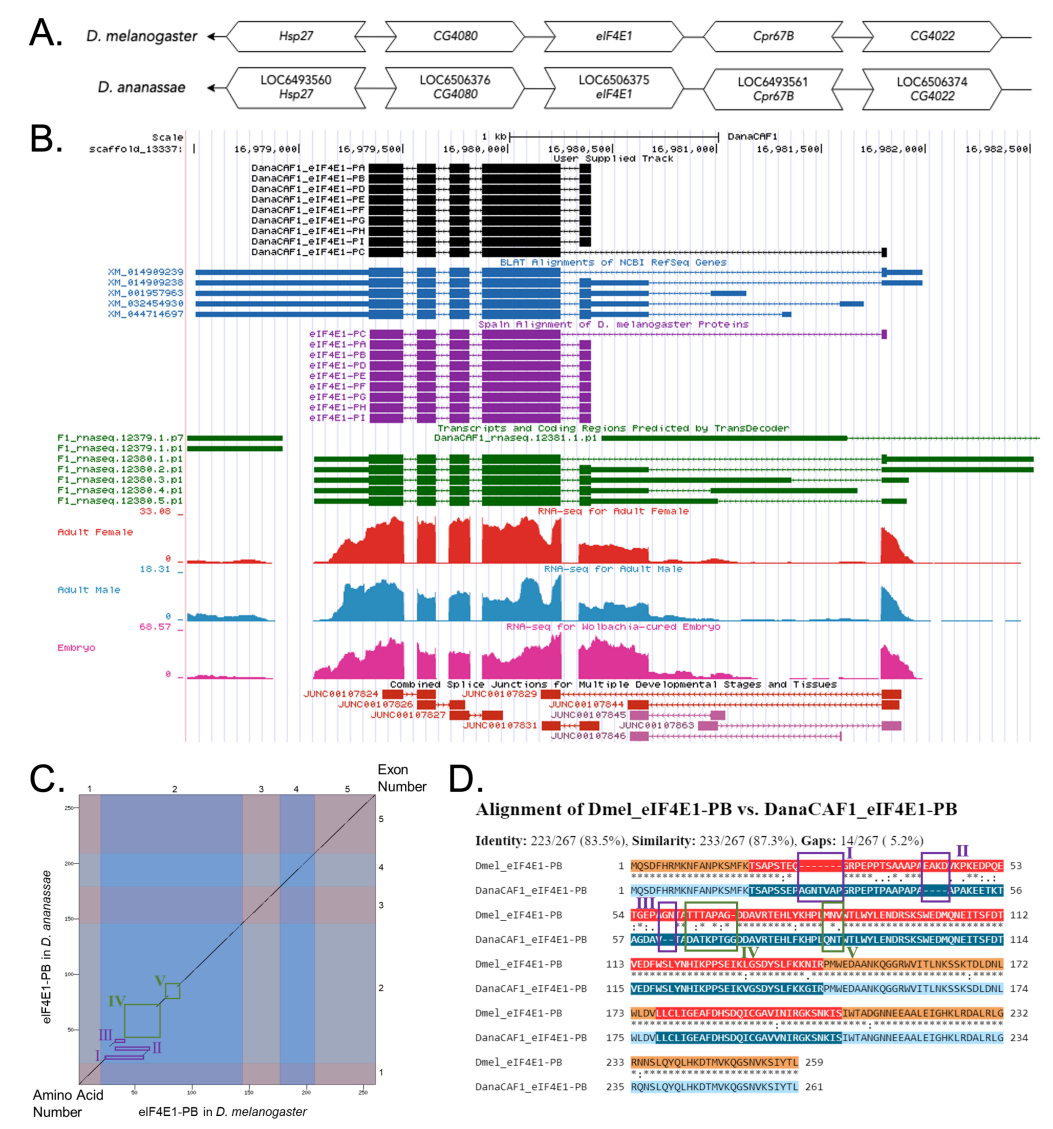
**
(A) Synteny comparison of the genomic neighborhoods for *eIF4E1* in *Drosophila melanogaster* and *D. ananassae* .** Thin underlying arrows indicate the DNA strand within which the gene– *eIF4E1* –is located in *D. melanogaster* (top) and *D. ananassae* (bottom) genomes. Thin arrows pointing to the left indicate that *eIF4E1* is on the negative (-) strand in *D. ananassae* and *D. melanogaster* . The wide gene arrows pointing in the same direction as *eIF4E1* are on the same strand relative to the thin underlying arrows, while wide gene arrows pointing in the opposite direction of *eIF4E1* are on the opposite strand relative to the thin underlying arrows. White gene arrows in *D. ananassae* indicate orthology to the corresponding gene in *D. melanogaster* . Gene symbols given in the *D. ananassae* gene arrows indicate the orthologous gene in *D. melanogaster* , while the locus identifiers are specific to *D. ananassae* . **(B) Gene Model in GEP UCSC Track Data Hub (Raney et al., 2014).** The coding-regions of *eIF4E1* in *D. ananassae* are displayed in the User Supplied Track (black); coding CDSs are depicted by thick rectangles and introns by thin lines with arrows indicating the direction of transcription. Subsequent evidence tracks include BLAT Alignments of NCBI RefSeq Genes (dark blue, alignment of Ref-Seq genes for *D. ananassae* ), Spaln of *D. melanogaster* Proteins (purple, alignment of Ref-Seq proteins from *D. melanogaster* ), Transcripts and Coding Regions Predicted by TransDecoder (dark green), RNA-Seq from Adult Females, Adult Males and Embryos (red, light blue, and dark pink, respectively; alignment of Illumina RNA-Seq reads from *D. ananassae* ), and Splice Junctions Predicted by regtools using *D. ananassae* RNA-Seq (SRP006203; SRP007906). Splice junctions shown have a minimum read-depth of 10 with 100-499 and >1000 supporting reads in light pink and red, respectively. **(C) Dot Plot of *eIF4E1-RB* in *D. melanogaster* ( *x* -axis) vs. the orthologous peptide in *D. ananassae* ( *y* -axis).** Amino acid number is indicated along the left and bottom; coding-CDS number is indicated along the top and right, and CDSs are also highlighted with alternating colors. The purple boxes labeled I, II, and III contain regions where an insertion or deletion of base pairs has occurred. The green boxes labeled IV and V outline regions that have a lack of sequence similarity between *eIF4E1-RB* in *D. melanogaster* and *eIF4E1-RB* in *D. ananassae* . **(D) The protein alignment between *D. melanogaster* eIF4E1-PB and its putative ortholog in *D. ananassae* .** The alternating colored rectangles represent adjacent CDSs. The symbols in the match line denote the level of similarity between the aligned residues. An asterisk (*) indicates that the aligned residues are identical. A colon (:) indicates the aligned residues have highly similar chemical properties—roughly equivalent to scoring > 0.5 in the Gonnet PAM 250 matrix (Gonnet et al., 1992). A period (.) indicates that the aligned residues have weakly similar chemically properties—roughly equivalent to scoring > 0 and ≤ 0.5 in the Gonnet PAM 250 matrix. A space indicates a gap or mismatch when the aligned residues have a complete lack of similarity—roughly equivalent to scoring ≤ 0 in the Gonnet PAM 250 matrix. The purple boxes labeled I, II and III outline regions where an insertion or deletion of base pairs has occurred. The green boxes labeled IV and V outline regions where there is a lack of sequence similarity between eIF4E1-PB in *D. melanogaster* and eIF4E1-PB in *D. ananassae* .

## Description

**Table:** 

*This article reports a predicted gene model generated by undergraduate work using a structured gene model annotation protocol defined by the Genomics Education Partnership (GEP; thegep.org) for Course-based Undergraduate Research Experience (CURE). The following information may be repeated in other articles submitted by participants using the same GEP CURE protocol for annotating Drosophila species orthologs of Drosophila melanogaster genes in the insulin signaling pathway.* "In this GEP CURE protocol students use web-based tools to manually annotate genes in non-model *Drosophila* species based on orthology to genes in the well-annotated model organism fruitfly *Drosophila melanogaster* . The GEP uses web-based tools to allow undergraduates to participate in course-based research by generating manual annotations of genes in non-model species (Rele et al., 2023) . Computational-based gene predictions in any organism are often improved by careful manual annotation and curation, allowing for more accurate analyses of gene and genome evolution (Mudge and Harrow 2016; Tello-Ruiz et al., 2019) . These models of orthologous genes across species, such as the one presented here, then provide a reliable basis for further evolutionary genomic analyses when made available to the scientific community.” (Myers et al., 2024) . “The particular gene ortholog described here was characterized as part of a developing dataset to study the evolution of the Insulin/insulin-like growth factor signaling pathway (IIS) across the genus *Drosophila* . The Insulin/insulin-like growth factor signaling pathway (IIS) is a highly conserved signaling pathway in animals and is central to mediating organismal responses to nutrients (Hietakangas and Cohen 2009; Grewal 2009) .” (Myers et al., 2024) . “ *eukaryotic translation initiation factor 4E1* ( *eIF4E1* ) encodes eIF4F cap-binding complex essential cap-dependent translation of mRNA, and binds the 7-methyl-guanosine cap structure of mRNA in *Drosophila* (Lachance et al., 2002; Lavoie et al., 1996) . The protein product of *eIF4E-3* , a paralog of *eIF4E1* , is specifically required during spermatogenesis in *Drosophila* (Hernendez et al., 2012).” (Lose et al., 2025) . “ *D* . *ananassae* (NCBI:txid7217) is part of the *melanogaster* species group within the subgenus *Sophophora* of the genus *Drosophila* (Sturtevant 1939; Bock and Wheeler 1972) . It was first described by Doleschall (1858). *D. ananassae* is circumtropical (Markow and O'Grady 2005; https://www.taxodros.uzh.ch, accessed 1 Feb 2023), and often associated with human settlement (Singh 2010) . It has been extensively studied as a model for its cytogenetic and genetic characteristics, and in experimental evolution (Kikkawa 1938; Singh and Yadav 2015) .” (Lawson et al., 2024) .

We propose a gene model for the *D. ananassae* ortholog of the *D. melanogaster* *eukaryotic translation initiation factor 4E1* ( *eIF4E1* ) gene. The genomic region of the ortholog corresponds to the uncharacterized protein LOC6506375 (RefSeq accession LOC6506375 ) in the Dana_CAF1 Genome Assembly of *D. ananassae* (GenBank Accession: GCA_000005115.1 ; Drosophila 12 Genomes Consortium et al., 2007). This model is based on RNA-Seq data from *D. ananassae* ( SRP006203 ; SRP007906 - Graveley et al., 2010) and *eIF4E1* in *D. melanogaster* using FlyBase release FB2022_04 ( GCA_000001215.4 ; Larkin et al., 2021; Gramates et al., 2022; Jenkins et al., 2022).

***Synteny***

The reference gene, *eIF4E1 ,* occurs on chromosome 3L in *D. melanogaster* and is flanked upstream by *CG4022* and *Cuticular protein 67B* ( *Cpr67B* ) and downstream by *CG4080* and *Heat shock protein 23* ( *Hsp23* ). The *tblastn* search of *D. melanogaster* eIF4E1-PB (query) against the *D. ananassae* (GenBank Accession: GCA_000005115.1 ) Genome Assembly (database) placed the putative ortholog of *eIF4E1* within scaffold scaffold_13337 ( CH902618.1 ) at locus LOC6506375 ( XP_014764725.1 ) — with an E-value of 8e-131 and a percent identity of 80.80%. Furthermore, the putative ortholog is flanked upstream by LOC6506374 ( XP_001958001.1 ) and LOC6493561 ( XP_001958000.1 ), which correspond to *CG4022* and *Cpr67B* in *D. melanogaster* (E-value: 6e-118 and 1e-168; identity: 88.17% and 97.31%, respectively, as determined by *blastp* ) ( Figure 1A, A ltschul et al., 1990). The putative ortholog of *eIF4E1* is flanked downstream by LOC6506376 ( XP_001957998.1 ) and LOC6493560 ( XP_001957997.1 ), which correspond to *CG4080* and *Hsp23* in *D. melanogaster* (E-value: 0.0 and 2e-103; identity: 87.56% and 83.08%, respectively, as determined by *blastp* ). The putative ortholog assignment for *eIF4E1* in *D. ananassae* is supported by the following evidence: The genes surrounding the *eIF4E1* ortholog are orthologous to the genes at the same locus in *D. melanogaster* and synteny is completely conserved, supported by results generated from *blastp* , so we conclude that LOC6506375 is the correct ortholog of *eIF4E1* in *D. ananassae* ( Figure 1A ).

***Protein Model***

*eIF4E1* in *D. ananassae* contains two unique protein-coding isoforms: eIF4E1-PB (identical to eIF4E1-PA, eIF4E1-PD, eIF4E1-PE, eIF4E1-PF, eIF4E1-PG, eIF4E1-PH, eIF4E1-PI) and eIF4E1-PC ( Figure 1B ). mRNA isoforms ( *eIF4E1-RB* , *eIF4E1-RA* , *eIF4E1-RD* , *eIF4E1-RE* , *eIF4E1-RF* , *eIF4E1-RG* , *eIF4E1-RH* , *eIF4E1-RI* ) contain five CDSs but differ in their UTRs. *eIF4E1-RC* differs from the other isoforms only in the first CDS. The remaining protein-coding isoforms are identical. Relative to the ortholog in *D. melanogaster* , the RNA CDS number and the protein isoform count are conserved. The sequence of eIF4E1-PB in *D. ananassae* has 80.80% identity (E-value: 8e-131) with the protein-coding isoform eIF4E1-PB in *D. melanogaster* , as determined by *blastp* ( Figure 1C ). Three indels were found within the second CDS of *D. ananassae* , outlined in boxes purple labeled I, II, and III ( Figure 1C, 1D ). Two regions containing a lack of sequence similarity were found between eIF4E1-PB in *D. ananassae* and *D. melanogaster* , outlined in green boxes labeled IV and V ( Figure 1C, 1D ). Coordinates of this curated gene model (eIF4E1-PE, eIF4E1-PF, eIF4E1-PH, eIF4E1-PA, eIF4E1-PG, eIF4E1-PI, eIF4E1-PD, eIF4E1-PB, eIF4E1-PC) are stored by NCBI at GenBank/BankIt (accessions BK064567 , BK064568 , BK064569 , BK064570 , BK064571 , BK064572 , BK064573 , BK064574 , and BK064575 , respectively). These data are also archived in the CaltechDATA repository (see “Extended Data” section below).

***Special characteristics of the protein model***

Three indels within CDS two, found in isoform *eIF4E1-RB* , are shown in the dot plot and protein alignment outlined by the purple boxes labeled I, II, and III ( Figure 1C and 1D). The first indel (I) consists of seven amino acids and is an insertion in *D. ananassae* and relative to *D. melanogaster* . The second (II) consists of four amino acids while the third (III) consists of two amino acids. Both (II and III) show deletions in *D. ananassae* relative to *D. melanogaster* .

## Methods

Detailed methods including algorithms, database versions, and citations for the complete annotation process can be found in Rele et al. (2023). Briefly, students use the GEP instance of the UCSC Genome Browser v.435 ( https://gander.wustl.edu ; Kent WJ et al., 2002; Navarro Gonzalez et al., 2021) to examine the genomic neighborhood of their reference IIS gene in the *D. melanogaster* genome assembly (Aug. 2014; BDGP Release 6 + ISO1 MT/dm6). Students then retrieve the protein sequence for the *D. melanogaster* reference gene for a given isoform and run it using *tblastn* against their target *Drosophila* species genome assembly on the NCBI BLAST server ( https://blast.ncbi.nlm.nih.gov/Blast.cgi ; Altschul et al., 1990) to identify potential orthologs. To validate the potential ortholog, students compare the local genomic neighborhood of their potential ortholog with the genomic neighborhood of their reference gene in *D. melanogaster* . This local synteny analysis includes at minimum the two upstream and downstream genes relative to their putative ortholog. They also explore other sets of genomic evidence using multiple alignment tracks in the Genome Browser, including BLAT alignments of RefSeq Genes, Spaln alignment of *D. melanogaster* proteins, multiple gene prediction tracks (e.g., GeMoMa, Geneid, Augustus), and modENCODE RNA-Seq from the target species. Detailed explanation of how these lines of genomic evidenced are leveraged by students in gene model development are described in Rele et al. (2023). Genomic structure information (e.g., CDSs, intron-exon number and boundaries, number of isoforms) for the *D. melanogaster* reference gene is retrieved through the Gene Record Finder ( https://gander.wustl.edu/~wilson/dmelgenerecord/index.html ; Rele et al *.,* 2023). Approximate splice sites within the target gene are determined using *tblastn* using the CDSs from the *D. melanogaste* r reference gene. Coordinates of CDSs are then refined by examining aligned modENCODE RNA-Seq data, and by applying paradigms of molecular biology such as identifying canonical splice site sequences and ensuring the maintenance of an open reading frame across hypothesized splice sites. Students then confirm the biological validity of their target gene model using the Gene Model Checker ( https://gander.wustl.edu/~wilson/dmelgenerecord/index.html ; Rele et al., 2023), which compares the structure and translated sequence from their hypothesized target gene model against the *D. melanogaster* reference gene model. At least two independent models for a gene are generated by students under mentorship of their faculty course instructors. Those models are then reconciled by a third independent researcher mentored by the project leaders to produce the final model. Note: comparison of 5' and 3' UTR sequence information is not included in this GEP CURE protocol.

## Data Availability

Description: A GFF, FASTA, and PEP of the model. Resource Type: Dataset. DOI: https://doi.org/10.22002/r7f2j-ads26
